# Patient safety culture and associated factors among nurses working at public hospitals in Gamo Zone, Southern Ethiopia

**DOI:** 10.1186/s12913-023-09671-6

**Published:** 2023-06-21

**Authors:** Bereket Beyene Shashamo, Gesila Endashaw Yesera, Meseret Girma Abate, Wubshet Estifanos Madebo, Lankamo Ena Digesa, Tamiru Chonka Choramo

**Affiliations:** 1 School of Nursing, Department of Comprehensive Nursing, College of Medicine and Health Sciences, Arba Minch University, P.O. Box 21, Arba Minch, Ethiopia; 2 School of Nursing, Department of Comprehensive Nursing, College of Medicine and Health Sciences, Arba Minch University, Arba Minch, Ethiopia; 3 School of Public Health, Department of Reproductive Health, College of Medicine and Health Sciences, Arba Minch University, Arba Minch, Ethiopia

**Keywords:** Patient safety, Patient safety culture, Gamo zone public hospitals, Nurses

## Abstract

**Background:**

Patient safety culture is the prevention of errors and adverse effects to patients associated with health care delivery. It is a vital component in the provision of quality care. In healthcare settings where there is a safety culture, the people (providers, staff, administrators, and patients/families) are engaged, encouraged, and supported to make care safer. Though it is an essential component in the provision of quality care, little is known about its level, contributory, and hindering factors from the nurses’ perspectives. This study aimed to assess patient safety culture and associated factors among nurses working at public Hospitals in Gamo Zone, Southern Ethiopia.

**Methods:**

This institution-based cross-sectional study was conducted among 398 nurses working at public hospitals in Gamo Zone. Data were collected by pretested, well-structured self-administered questionnaire from June 1 to 30, 2022. The collected data were checked, coded, and entered into Epi-data version 4.6.0.2 and were exported to SPSS version 25 for analyses. Bivariable and multivariable logistic regression was done to identify independent factors associated with patients’ safety culture.

**Results:**

This study revealed that 202(50.8%), 95% CI: (46%—56%) of the participants had indicated good patient safety culture. From factors analysis, having an educational status of a bachelor’s degree and above [AOR = 2.26, 95%CI: (1.13—4.52)], working in a surgical ward [AOR = 5.48, 95%CI: (1.96—15.34)], not being blamed when medical errors happened [AOR = 3.60, 95%CI: (1.82 – 7.14)], and working 40 up to 49 h per week [AOR = 0.30, 95%CI: (0.13 – 0.74)] were identified to be significantly associated with good patient safety culture.

**Conclusion:**

Based on the study findings, it could be observed that good patient safety culture was indicated only by half of the study participants. Implementing actions that support dimensions of patient safety culture, and creating opportunities for continuous educational advancement is recommended. Moreover, Hospital administrators, nurses’ directors, and healthcare policy-makers should work in collaboration to improve the patient safety culture, and also it would be better to create a blame-free environment to promote event reporting practices.

## Background

Patient safety is the prevention of errors and adverse effects on patients associated with health care [1]. Patient safety culture is the extent to which an organization's culture supports and promotes patient safety and refers to the beliefs, values, and norms that are shared by healthcare practitioners and other staff throughout the organization that influences their actions and behaviours [2]. It can be measured by determining what is rewarded, supported, expected, and accepted in an organization as it relates to patient safety [2]. An event is any type of error, mistake, incident, accident, or deviation, regardless of whether or not it results in patient harm [2].

Globally, it is estimated that 421 million hospitalizations take place in the world annually, and approximately 42.7 million adverse events occur in patients during those hospitalizations from them two-thirds of all adverse events happen in low and middle-income countries [3]. Unsafe health care through its adverse events is likely to be one of the 10 leading causes of death and disability worldwide [4]. Evidence suggests that 134 million adverse events occur each year due to unsafe care in hospitals in low and middle-income countries (LMICs), resulting in 2.6 million deaths annually [5].

Patient safety is a serious global public health concern. In high-income countries, as many as one in 10 patients were harmed while receiving hospital care [6, 7]. And it was stated that these harms can be caused by a range of adverse events, with nearly 50% of them can be considered preventable [8]. A study on the frequency and preventability of adverse events across 26 hospitals in eight low and middle-income countries, showed the adverse event rate to be around 8%. Of these events, 83% were preventable, while about 30% were associated with death of the patient [9, 10].

The safety of health care is now a major global concern and services that are unsafe and of low-quality lead to diminished health outcomes and even harm [11]. Assessing and promoting the culture of patient safety is recognized as a prerequisite step toward improving patient safety [12]. Even though the diversity of the population is affecting the healthcare delivery system, nurses play a major role in the delivery of healthcare through close interaction with patients 24 h per day, the highest contact frequency, providing direct care to patients and families, spending maximum time with the patients and their families, and providing constant emotional, spiritual, and personal support [13]. In addition to that, nurses play a significant role in framing the healthcare delivery system and its strong influence on patients’ outcomes [14].

Moreover, by having a better understanding of the present healthcare condition of a certain organization, nurses would be able to identify areas in the healthcare system that need more or immediate attention to reduce faults and lapses in delivering safe and quality care to patients [14]. Therefore, assessing their perspective towards the patient safety culture is very important to be considered and utilized.

A study has revealed that not enhancing patient safety culture can increase the number of missed nursing care and adverse patient outcomes [15]. Furthermore, the studies demonstrated that there was a negative attitude towards patient safety among nurses [16, 17]. And this absence of positive perception toward patient safety culture among nurses can affect their adherence to standard precautions and may result in negative health outcomes among patients [18]. In addition to that, it was known that impairment in patient safety culture can expose nurses to a high level of job-related stress which may also have a negative outcome on the health care for patients [19].

Though studies were conducted in different parts of the world, the researchers have observed gaps to be filled in terms of perspectives, population, implication, and settings variations. Coming to the Ethiopian setting, even though the concern is given to patients’ safety culture, a significant number of patients are suffering from inadequately ensured safety culture. Most of the findings were from the perspectives of non-clinical staff, community pharmacists, health science students, and health care providers from different specialties in a general way. From this, we can see that there is an observable lack of findings from nurses’ perspectives. Hence, nurses have a big chance of having a long time of contact with patients, the findings from their perspective are very vital to ensure patient safety. Moreover, to the level of investigators’ knowledge, this study is the first to assess the current state of patient safety culture from the nurses’ perspective in Ethiopia in general and in the study area specifically. Therefore, this study addresses these gaps by assessing patient safety culture and associated factors at public Hospitals in Gamo Zone, Southern Ethiopia from nurses’ perspective.

## Methods

### Study setting and period

This study was conducted in public hospitals of Gamo Zone, Southern Ethiopia from June 1 to 30, 2022. Gamo zone is located in southern nation nationalities and people’s regional state, Southern Ethiopia. This Zone hosted different general and primary hospitals, which serve the community by providing preventive and curative services. During the conduction of this study, this zone has six public hospitals. Namely: Arbaminch general hospital, Dil fana primary hospital, Chencha primary hospital, Gerese Primary Hospital, Kamba primary hospital, and Selamber primary hospital.

### Study design

An institution-based cross-sectional study design was employed.

### Study population

All nurses who are working at public hospitals in Gamo Zone during the data collection period and fulfill the inclusion criteria.

### Inclusion and exclusion criteria

All nurses who were staff in each hospital and have work experience of at least six months were included in this study. Whereas those nurses who were on annual leave during the data collection period were excluded.

### Sample size determination

The sample sizes for all specific objectives were computed independently and the larger sample size was taken. Accordingly, the sample size for the first specific objective (to assess the magnitude of patient safety culture) was calculated using a single population proportion formula using the following assumptions: proportion of good patient safety culture (44%) from a previous study [20], 95% confidence level, 5% margin of error, and 10% non-response rate. And the sample size was 417.

The sample size of the second specific objective (to identify factors associated with patient safety culture) was calculated by considering the factor that was significantly associated with the patient safety culture in a previous study [21], 95% confidence level, 5% margin of error, power of 80%, and the ratio of exposed to unexposed 1:1 using STAT CALC of Epi-info Version 7 and 10% non-response rate was added to the initial sample. And the sample size was 178. Finally, the required sample size for this study was decided to be 417 by taking the larger sample size from the two ample sizes (i.e. calculated for the first specific objective = 417) and the second specific objective = 178).

### Sampling procedure

All nurses who were working at public hospitals in Gamo Zone and fulfilled the inclusion criteria were included in this study. During the data collection period, the total number of nurses working at public hospitals in Gamo Zone was 408. But the calculated sample size was 417. We included all nurses in this study to increase its power.

### Data collection tool

The data were collected using a pre-tested, well-structured, self-administered questionnaire. The items for assessing patient safety culture were standardized and validated. It has been widely validated, tested in different settings, and used widely in different parts of the world, and recent studies conducted in Ethiopia [20–25]. The Hospital Survey on Patient Safety Culture (HSOPSC) tool includes 42 items that measure 12 dimensions or composites of patient safety culture: “Teamwork within hospital units” (4 items), “Supervisor/manager expectations and actions promoting safety” (4 items), “Organizational learning–continuous improvement” (3 items), “ Hospital management support for patient safety” (3 items), “Overall perceptions of patient safety” (4 items), ‘Communication openness’ (3 items), “Teamwork across hospital units/departments” (4 items), “Staffing” (4 items), “Handoffs and transitions” (4 items), “Non-punitive response to error” (3 items), “Feedback and communication about errors’ (3 items), and “Frequency of events reported” (3 items), The response to each item in the questionnaire was assessed using a 5-point Likert scale of agreement (from Strongly disagree = 1; Disagree = 2; Neither = 3; Agree = 4; Strongly agree = 5) or 5-point frequency scale (from Never = 1; Rarely = 2; Sometimes = 3; Most of the time = 4; Always = 5). The tool included both positively and negatively worded items for agreement. There were also items to assess the frequency of event reporting, feedback, and communication about errors. Agree or strongly agree were considered as positive responses to positively worded items, and disagree or strongly disagree were considered as positive responses to negatively worded items. Negatively worded items were reverse coded so that a higher score would indicate a more positive response. For the frequency of events reporting and feedback and communication about errors dimensions, most of the time or always were considered as positive responses. These positive responses were added. Then, the level of patient safety culture was categorized as good or poor, with scores of ≥ 75% and < 75%, respectively [21]. The questionnaire also has parts for assessing socio-demographic characteristics and healthcare system-related and work-related variables.

### Data collectors and data collection procedures

A well-trained six diploma nurses facilitated the data collection process and six B.Sc. nurses supervised the data collection process. They were given information about the study's aim and the possible procedures to follow before and during the data collection process. Both facilitators and supervisors were recruited from other hospitals rather than the hospitals in which they are working. Facilitators informed the nurses about the purpose of the research. The nurses were encouraged to feel free and were told that the confidentiality of their responses will be assured and their names and their hospital name will not be written on the questionnaire. After this, nurses who were willing to participate and signed the informed, voluntary written consent document were given the questionnaire after they had finished their duty hours.

### Study variables

Patient Safety Culture was the dependent variable, and Socio-demographic characteristics of the nurses, health care system-related and work-related characteristics were independent variables in this study.

### Data quality control

Hence English is the media of instruction in all Ethiopian nursing schools, the English version of the questionnaire was used. A pre-test in 5% of the sample was conducted in the Hospital out of the study area (i.e. Jinka General Hospital) before the actual data collection period. By doing that inconsistencies and misunderstandings of the questions to assure reliability were checked. Extensive training for two days was given to facilitators and supervisors on the objectives of the study, data collection tool, questionnaires, ways of conducting facilitation, checking the completeness of data collection tools, and how to maintain confidentiality. The collected data were reviewed and checked for legibility of handwriting, completeness, and consistency by the principal investigator and supervisors. Investigators and supervisors monitored the process of data collection on a daily base. Before entering the data into the software, the data were checked for completeness once again by the data clerk. Proper coding and categorization of the variables were maintained during the data entry.

### Data processing and analyses

The collected data were coded and entered into Epi data version 4.6.0.2. Then, the data were exported to SPSS window version 25 for analysis. Descriptive analyses such as simple frequencies, measures of central tendency, and measures of variability were used to describe the characteristics of participants. The assumptions for binary logistic regression were checked. And bivariable analyses were done and independent variables that yield a *p*-value of  ≤ 0.25 were included in the multivariable analysis. Hosmer–Lemeshow statistic and Omnibus tests were done for checking model fitness. Multi-collinearity was checked through Collinearity statistics (Variance inflation factor (VIF) > 10 and tolerance (T) < 0.1 were considered suggestive of the existence of multi-co-linearity). Adjusted odds ratios with 95% CI were estimated to identify the factors associated with patient safety culture. The level of statistical significance was declared at *p*-value ≤ 0.05.

## Results

### Socio-demographic characteristics of the study participants

Out of 408 expected participants, 398 participated in this study making a response rate of 97.55%. Among the respondents, 205(51.5%) were females. The mean age was 35.77 (SD ± 6.569) years and more than half of the respondents, 262(65.8%) lie between the 30–34 age group. Regarding their educational status, the larger proportion of the participants, 265 (66.6%) had a bachelor’s degree and above. More than half of them 221(55.5%) were married (Table 1).

**Table 1:** Socio-demographic characteristics of the study participants (*N* = 398)

Characteristics	Category	Frequency (N)	Percent (%)
Sex	Male	193	48.5
	Female	205	51.5
Age in years	≤ 29 years	96	24.1
	30 – 44 years	262	65.8
	≥ 45 years	40	10.1
Educational status	Diploma	133	33.4
	BSc & above	265	66.6
Marital status	Single	150	37.7
	Married	221	55.5
	Divorced/Widowed	27	6.8
Monthly salary in birr	≤ 6000	84	21.1
	> 6000	314	78.9

### Healthcare system and work-related characteristics of the study participants

Nearly half, 188(47.2%) of the participants had work experience of ≤ 5 years at their hospital. Regarding the working unit distribution of the respondents, 167 (42.0%) of them were working in the surgical ward. This study found that 211(53.0%) of nurses had reported the presence of workload. More than half of the participants, 238 (59.8%) had indicated that they had reported adverse events (Table 2).

**Table 2:** Frequency and percentage distribution of health care system and work-related characteristics of the study participants

Characteristics	Category	Frequency (N)	Percent (%)
Hospital type	Primary	217	54.5
	General	181	45.5
Years of work experience at the hospital	≤ 5 years	188	47.2
	6–10 years	124	31.2
	≥ 11 years	86	21.6
Working unit	Medical ward	71	17.8
	Surgical ward	167	42.0
	Pediatrics	63	15.8
	OPD	49	12.3
	Others^a^	48	12.1
Years of experience in the current unit	≤ 5 years	291	73.1
	6–10 years	82	20.6
	≥ 11 years	25	6.3
Perceived level of job satisfaction	Satisfied	234	58.8
	Not satisfied	164	41.2
Received training	Yes	217	54.5
	No	181	45.5
Type of Shifting	Every 8 h	236	59.3
	Regular (day)	162	40.7
Participation in the patient safety program	Yes	247	62.1
	No	151	37.9
Ever reported adverse event	Yes	238	59.8
	No	160	40.2
Management encourages reporting an adverse event	Yes	250	62.8
	No	148	37.2
Management blame when medical errors happened	Yes	155	38.9
	No	243	61.1
Hours worked per week	40–59 h	133	33.4
	60–79 h	190	47.7
	≥ 80 h	75	18.8
Get necessary equipment/ materials timely at the time of giving care	Yes	266	66.8
	No	132	33.2
Workload	Yes	211	53.0
	No	187	47.0

Others^a^ = Operation room (OR), Emergency, Gyn & Obs ward, ICU, EPI, Dental, and Triage

### Level of patient safety culture

**Fig. 1:**
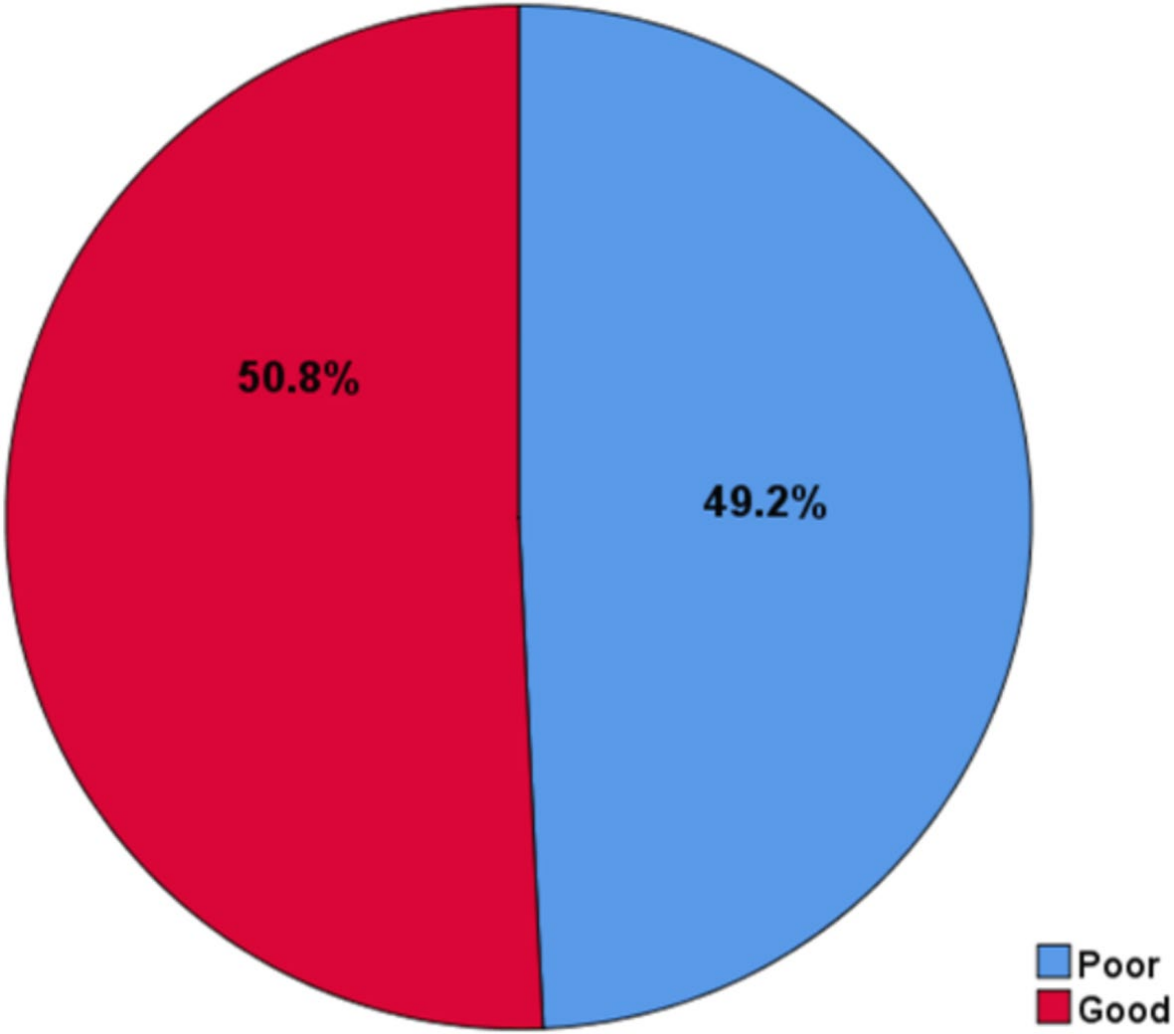
The Level of Patient Safety Culture in Public Hospitals of Gamo, Zone, Southern Ethiopia 2022

The level of patient safety culture was computed from the score for patient safety culture-related questions and the sum score of 75% and above was considered as there is a good patient safety culture and the sum score of below 75% was considered as there is poor patient safety culture. According to this, half (202(50.8%), 95% CI: (46%—56%)) of the participants had indicated good patient safety culture (Fig. 1).

Regarding the average percentage of positive scores on patient safety-culture components, hospital handoffs, and transition (68.03%), communication openness (67.54%), teamwork within hospital units (67.48%), and organizational learning- continuous improvement (66.90%) were the highest positively contributing dimensions for overall patient safety culture. Whereas, frequency of event reporting (57.47%) was the lowest average percentage of positive scores (Table 3).

**Table 3:** Patient safety culture dimensions at public hospitals of Gamo Zone, Southern Ethiopia 2022 (*N* = 398)

S. no	Patient safety culture dimensions	Num ber of items	Positive patient safety culture score (%)
1	Hospital handoffs and transition	4	68.03
2	Communication openness	3	67.54
3	Teamwork within hospital units	4	67.48
4	Organizational learning- continuous improvement	3	66.90
5	Teamwork across hospital units/departments	4	66.65
6	The overall perception of patient safety	4	66.58
7	Staffing	4	65.45
8	Non-punitive response to the error	3	65.07
9	Supervisor/manager expectations and actions promoting safety	4	63.95
10	Feedback and communication about errors	3	63.40
11	Hospital management support for patient safety	3	58.37
12	Frequency of event reporting	3	57.47

### Factors associated with patient safety culture

In bivariable analyses, patient safety culture was significantly associated with sex, age, educational status, marital status, total years of work experience, current working unit, satisfaction level, attendance of training, participation in the patient safety program, reporting an adverse event, encouragement for reporting of an adverse event, blame when medical errors happened, hours worked per week, getting necessary equipment/materials timely at the time of giving care and workload. On the contrary, there was no association between patient safety culture and monthly salary, type of hospital, current working unit experience, and type of shifting (Table 4).

**Table 4:** Bivariable analyses of the factors associated with patent safety culture

Variables	Category	Patient safety culture		COR (95% CI)
		Good	Poor	
Sex	Male	84	109	1.00
	Female	118	87	1.76 (1.18–2.62)*
Age in years	≤ 29 years	22	74	1.00
	30 – 44 years	159	103	5.19 (3.04–8.88)*
	≥ 45 years	21	19	3.72 (1.70–8.13)*
Educational level	Diploma	36	97	1.00
	BSc & above	166	99	4.52 (2..86–7.13)*
Marital status	Single	39	111	1.00
	Married	145	76	5.43 (3.43–8.59)*
	Divorced/Widowed	18	9	5.69 (2.36–13.7)*
Monthly salary in birr	≤ 6000	41	43	1.00
	> 6000	161	153	1.10 (0.68–1.79)
Type of hospital	Primary	112	105	1.00
	General	91	90	0.93 (0.63–1.38)
Years of work experience at the hospital	≤ 5 years	52	136	1.00
	6 – 10 years	87	37	6.15 (3.73–10.14)*
	≥ 11 years	63	23	7.16 (4.03–12.73)*
Working unit	Medical ward	12	59	1.00
	Surgical ward	134	33	19.97 (9.64–41.36)*
	Pediatrics	27	36	3.69 (1.66–8.18)*
	OPD	20	29	3.39 (1.46–7.87)*
	Others^a^	9	39	1.14 (0.44–2.95)
Years of experience in the current unit	≤ 5 years	142	149	1.00
	6 – 10 years	48	34	1.48 (0.90–2.43)
	≥ 11 years	12	13	0.97 (0.43–2.20)
Job satisfaction	Satisfied	142	92	2.68 (1.77–4.04)*
	Not satisfied	60	104	1.00
Received training	Yes	134	83	2.68 (1.79–4.03)*
	No	68	113	1.00
Type of shifting	Every 8 h	120	116	1.01 (0.68–1.51)
	Regular (day)	82	80	1.00
Participation in the patient safety program	Yes	150	97	2.94 (1.93–4.49)*
	No	52	99	1.00
Ever reported adverse event	Yes	148	90	3.23 (2.12–4.91)*
	No	54	106	1.00
Management encourages reporting of an adverse event	Yes	157	93	3.86 (2.50–5.96)*
	No	45	103	1.00
Management blame when medical errors happened	Yes	38	117	1.00
	No	164	79	6.39 (4.06–10.06)*
Hours worked per week	40–59 h	46	87	0.94 (0.52–1.70)
	60–79 h	129	61	3.76 (2.15–6.60)*
	≥ 80 h	27	48	1.00
Get necessary equipment/ materials timely at the time of giving care	Yes	160	106	3.24 (2.08–5.03)*
	No	42	90	1.00
Workload	Yes	71	140	1.00
	No	131	56	4.61 (3.02–7.05)*

1 = reference, Others^a^ = Operation room (OR), Emergency, Gyn & Obs ward, ICU, EPI, Dental, and Triage, * = Significant at *P*-value ≤ 0.05, *COR* Crude Odd Ratio, *CI* Confidence Interval

In multivariable analyses, from the variables which showed significant association in bivariable analyses, educational status, current working unit, blame when medical errors happened, and hours worked per week were identified to be significantly associated with patient safety culture.

According to this study, the odds of having good patient safety culture were 2.3 times [AOR = 2.26, 95%CI: (1.13—4.52)] higher among nurses who have a bachelor’s degree and above when compared to the nurses who have a diploma. Participants who were working in the surgical ward were found to have 5.5 times [AOR = 5.48, 95%CI: (1.96—15.34)] higher odds of having good patient safety culture when compared to the participants working in the medical ward.

In this study, the participants who were not blamed when medical errors happened were known to have 3.6 times [AOR = 3.60, 95%CI: (1.82 – 7.14)] more odds to indicate good patient safety culture. This study also found that the participants who had worked 40–49 h per week were revealed to have 0.3 times [AOR = 0.30, 95%CI: (0.13 – 0.74)] fewer odds of having good patient safety culture when compared to the participants who had worked ≥ 80 h per week (Table 5).

**Table 5:** Multivariable analyses of the factors associated with patent safety culture (*N* = 398)

Variables	Category	Patient safety culture		COR (95% CI)	AOR (95% CI)
		Good	Poor		
Sex	Male	84	109	1.00	1.00
	Female	118	87	1.76 (1.18–2.62)*	0.87 (0.48–1.56)
Age in years	≤ 29 years	22	74	1.00	1.00
	30 – 44 years	159	103	5.19 (3.04–8.88)*	1.54 (0.61–3.91)
	≥ 45 years	21	19	3.72 (1.70–8.13)*	0.60 (0.17–2.15)
Educational level	Diploma	36	97	1.00	1.00
	BSc & above	166	99	4.52 (2..86–7.13)*	2.26 (1.13–4.52)*
Marital status	Single	39	111	1.00	1.00
	Married	145	76	5.43 (3.43–8.59)*	1.41 (0.57–3.49)
	Divorced/Widowed	18	9	5.69 (2.36–13.7)*	2.35 (0.62–8.87)
Years of work experience at the hospital	≤ 5 years	52	136	1.00	1.00
	6 – 10 years	87	37	6.15 (3.73–10.14)*	1.85 (0.90–3.83)
	≥ 11 years	63	23	7.16 (4.03–12.73)*	1.77 (0.75–4.19)
Working unit	Medical ward	12	59	1.00	1.00
	Surgical ward	134	33	19.97 (9.64–41.36)*	5.48 (1.96–15.34)*
	Pediatrics	27	36	3.69 (1.66–8.18)*	1.68 (0.58–4.90)
	OPD	20	29	3.39 (1.46–7.87)*	2.42 (0.81–7.30)
	Others^a^	9	39	1.14 (0.44–2.95)	0.64 (0.19–2.15)
Job satisfaction	Satisfied	142	92	2.68 (1.77–4.04)*	0.61 (0.31–1.23)
	Not satisfied	60	104	1.00	1.00
Received training	Yes	134	83	2.68 (1.79–4.03)*	0.69 (0.34–1.40)
	No	68	113	1.00	1.00
Participation in the patient safety program	Yes	150	97	2.94 (1.93–4.49)*	0.90 (0.42–1.90)
	No	52	99	1.00	1.00
Ever reported adverse event	Yes	148	90	3.23 (2.12–4.91)*	1.21 (0.61–2.38)
	No	54	106	1.00	1.00
Management encourages reporting an adverse event	Yes	157	93	3.86 (2.50 –5.96)*	1.47 (0.73–2.96)
	No	45	103	1.00	1.00
Management blame when medical errors happened	Yes	38	117	1.00	1.00
	No	164	79	6.39 (4.06 –10.06)*	3.60 (1.82–7.14)*
Hours worked per week	40–59 h	46	87	0.94 (0.52–1.70)	0.30 (0.13–0.74)*
	60–79 h	129	61	3.76 (2.15–6.60)*	1.96 (0.86–4.45)
	≥ 80 h	27	48	1.00	1.00
Get the necessary equipment at the time of giving care	Yes	160	106	3.24 (2.08–5.03)*	1.26 (0.64–2.50)
	No	42	90	1.00	1.00
Workload	Yes	71	140	1.00	1.00
	No	131	56	4.61 (3.02–7.05)*	1.67 (0.92–3.01)

1 = reference, Others^a^ = Operation room (OR), Emergency, Gyn & Obs ward, ICU, EPI, Dental, and Triage, * = Significant at *P*-value ≤ 0.05, *COR* Crude Odd Ratio, *CI* Confidence Interval, *AOR* Adjusted Odd Ratio

## Discussion

This study was conducted among nurses working at public hospitals in Gamo Zone, Southern Ethiopia, to assess patient safety culture perceptions among nurses and explore factors associated with patient safety culture. Hence, patient safety culture is a vital element in creating patient safety in healthcare organisations. The findings of this study could contribute valuable information towards improving the quality of healthcare for patients. With this information, the administrative bodies of medical care institutions will be able to determine aspects that need attention; areas of weakness, and strengths associated with patient safety culture and will take appropriate actions as needed. Ultimately, it provides information that will help improve patient-related outcomes in the healthcare delivery process.

The magnitude of good patient safety culture indicated in this study is comparable with the results of studies conducted in Asia (53.58%) [26], in Europe (53.9%) [27], in Jordan (which ranges from 21 up to 78.8%) [28], in Oman (which ranges from 21.4 up to 83.4%) [29], and in Ethiopia which ranges from 46% to 49.2% [22, 24, 25]. It is lower than the result of the study conducted in Spain (62%) [30], in four countries of Europe (61.3%) [31], in China (61.3%) [32], in Egypt (88.36%) [33]. Whereas it is higher than the findings of previous studies conducted in Iran (34.1%) [34], Egypt (30%) [35], Malaysia (23.9%) [36], and Ethiopia which ranges from 44% up to 45.3% [20, 21, 23]. The possible reason for this difference may be due to the differences in infrastructural and economic status among settings, and also the management and organizational leadership behaviour, and relationships among hospital staff can contribute to this difference.

In this study hospital handoffs and transition, communication openness, and teamwork within hospital units were the areas with the highest average positive response rate of 68.03%, 67.54%, and 67.48% respectively. This implies that respondents are positive in passing on important patient care information during shift changes, freely speaking up if they see something that may negatively affect patient care, supporting one another in transferring patients from one unit to another, working together as a team, and doing things to improve patient safety. This is supported by the previous studies conducted in Brazil and Portuguese nurses [37], and in Ethiopia [20, 21, 23, 24]. Also, regarding teamwork within hospital units, this study's finding is in line with a previous study conducted in Japan [38]. But it is in contrast with the study conducted in Amhara Region [25]. Which found that Handoffs and transitions as major safety problems. The possible reason for this difference may be due to the time gap and variation in setting from place to place. In this study, the frequency of event reporting was the areas with the lowest average positive response rate of (57.47%). This finding is supported by the previous study conducted in Switzerland [39], in Australia [40].

This study finding revealed that there were higher odds of having good patient safety culture among respondents who have a bachelor’s degree and above. This implies that advancement in educational status has a positive effect on the improvement of patient safety culture. This is supported by the study conducted in Gondar [23]. That found that master’s degree holders have higher patient safety culture scores than those healthcare professionals with bachelor's degree holders. Also, it is in line with a previous study conducted in Malaysia [36]. That study found that the increment in educational status has a positive and significant effect on patient safety culture. This can be due to the reason that when nurses advance their educational status it could help them to get more knowledge on how to maintain good patient safety culture through different techniques and as a result of that they could be more capable of maintaining a good patient safety culture But it is in contrast to the study conducted in Dessie [21]. This found that educational status was not significantly associated with patient safety culture.

This study found that the current working unit is significantly associated with patient safety culture. This is consistent with the findings of previous studies conducted in Sweden [41], and in Ethiopia Dessie [21]. Participants who were working in the surgical ward had higher odds of having good patient safety culture. This implies that there is a positive relationship between working in the surgical ward and good patient safety culture. This can be due to the reason that the nature of care which is delivered to the patients admitted in the surgical ward may necessitate teamwork, frequent communication, and support of one another among the professionals within the same unit, across the different hospital units, and between the management bodies and professionals. Therefore, nurses assigned and working in the surgical ward may have higher odds of indicating good patient safety culture through different mechanisms.

In this study, the participants who were not being blamed when medical errors happened were known to have more odds to indicate good patient safety culture. This implies that making the nurse prone to unnecessary blame when medical errors happened hurts the patient safety culture. This is supported by the study conducted in Bale [20]. Which found not providing a conducive work climate and holding the event reports against care providers only, and keeping it in their files had affected patient safety culture. Again, it is consistent with the findings of a previous study conducted in Malaysia [36], and South Korea [42]. Those studies found that creating a non-blaming and supportive working environment has a positive and significant effect on patient safety culture. This can be due to the reason that when nurses are blamed when medical errors happened they could suffer from shame, and lack of team spirit, and this could affect their ability to maintain good patient safety. As a result of that the dimensions of patient safety culture could be compromised. Therefore, they could have fewer odds of indicating a good patient safety culture.

The finding of this study indicated that hours worked per week are significantly associated with patient safety culture. This is consistent with previous studies conducted in Ethiopia [20, 22]. This study also found that the participants who had worked 40–49 h per week had fewer odds of having good patient safety culture. This indicates that staying for a short period in the hospital exerts a negative effect on the patient safety culture. This is supported by the study conducted in Bale [20]. But it is in contrast with the study conducted in Jimma [22]. This revealed that for every unit increase in hours worked per week, the patient safety culture score was found to reduce. This can be due to the reason that if nurses stay for a long period in the hospital, they may build team spirit, and communication on their care, share feedback from one another about their care regarding the challenges and achievements, find areas for improvement, and act accordingly. This could result in higher odds of indicating a good patient safety culture.

As per this study, sex, age, marital status, Years of work experience at the hospital, satisfaction level, attendance of training, participation in the patient safety program, reporting an adverse event, encouragement for reporting of an adverse event, getting necessary equipment/materials timely at the time of giving care and workload were not significantly associated with patient safety culture. Regarding years of work experience at the hospital, this result is in line with the study conducted in Dessie [21]. But it is in contrast with the study conducted in Jimma [22]. Regarding age, sex, and participation in the patient safety program it is not consistent with a previous study conducted in South East Asia [36]. That study found that age, sex, and participation in the patient safety program has a positive and significant effect on patient safety culture. The possible reason for this difference may be due to the differences in methodological approaches and variation in setting from place to place. Also, it can be due to the differences in the way of categorizing variables.

This study revealed the recent status of patient safety culture and its associated factors. This has public importance and gives information to do interventions on the dimensions of patient safety culture to improve the patient’s safety during the healthcare delivery process. The present study used widely validated data collection tools tested in different settings. However, there is a possibility of social-desirability bias and there may be the possibility of over-reporting. But the effort was made to minimize it through a genuine explanation of the objectives and significance of the study, and by including negative items. Moreover, the findings of this study cannot be applied to other settings but can be applied only to public hospitals.

## Conclusions

This study provides information about the magnitude of patient safety culture and its associated factors at public hospitals in Gamo Zone, southern Ethiopia, from the nurses’ perspective. The results demonstrate the low magnitude of a good patient safety culture, contributing to identifying areas requiring enhancement and factors impeding the development of a good patient safety culture. The hospital handoffs and transition dimension received the highest positive patient safety culture score, and the frequency of event reporting dimension received the lowest positive patient safety culture score among the participants. In terms of educational status, participants with lower educational status showed lower odds of having a good patient safety culture than the more educated participants. Participants working in surgical wards showed higher odds of a good patient safety culture than those working in medical wards. Ensuring a blame-free working environment was known to increase the odds of having a good patient safety culture. Participants who worked 40–49 h per week showed lower odds of having a good patient safety culture than those working ≥ 80 h.

## Data Availability

The data sets used/or analyzed during the current study are available from the corresponding author on reasonable request.

## Abbreviations

AHRQ: Agency of Healthcare Research and Quality
AOR: Adjusted Odd Ratio
CI: Confidence Interval
COR: Crude Odd Ratio
EPI: Expanded Program on Immunization
ETB: Ethiopian Birr
FMH: Federal Ministry of Health
HSOPSC: Hospital Survey on Patient Safety Culture
ICU: Intensive Care Unit
IRB: Institutional Research Ethics Review Board
LMICs: Low and middle-income countries
OPD: Out Patient Department
OR: Operation room
SNNPR: Southern Nations Nationalities and Peoples Region
WHO: World Health Organization
